# Dermatopathology and the Diagnosis of Fungal Infections

**DOI:** 10.3389/bjbs.2023.11314

**Published:** 2023-06-07

**Authors:** S. A. Howell

**Affiliations:** Mycology, St John’s Specialist Dermatology Laboratories, Synnovis, London, United Kingdom

**Keywords:** dermatopathology, fungal infection, tissue biopsy, histopathology, mycoses

## Abstract

Diagnosis of superficial/cutaneous fungal infections from skin, hair and nail samples is generally achieved using microscopy and culture in a microbiology laboratory, however, any presentation that is unusual or subcutaneous is sampled by taking a biopsy. Using histological techniques a tissue biopsy enables a pathologist to perform a full examination of the skin structure, detect any inflammatory processes or the presence of an infectious agent or foreign body. Histopathological examination can give a presumptive diagnosis while a culture result is pending, and may provide valuable diagnostic information if culture fails. This review demonstrates how histopathology contributes to the diagnosis of fungal infections from the superficial to the life threatening.

## Introduction

The prevalence of fungal infection is far greater than might be thought. Fungal infections of skin, hair and nail have been estimated to affect one to two billion people worldwide, mucosal *candida* infections affect tens of millions of people, and serious fungal infections affect 150 million people (1, 2). Mortality from the serious fungal infections has been estimated to be similar to the number of deaths from tuberculosis (1). Superficial fungal infections have been reported to affect 20%–25% of the world’s population with dermatophyte infections predominating (3). Global estimation of the prevalence of subcutaneous infections is difficult as these infections are less common, some are more prevalent in certain areas or regions, and are not notifiable diseases. Therefore, subcutaneous fungal infections are under reported and are considered to be Neglected Tropical Diseases by the World Health Organisation (4). Systemic infections involve the internal organs and may disseminate to the skin *via* the blood. Many systemic infections occur in immune compromised patients and the prevalence of these infections varies according to patient risk factors, country or region, and availability of surveillance data (1).

The diagnosis of cutaneous fungal infections requires laboratory testing with microscopic examination of tissue to detect the presence of a fungus, and culture to enable the identification of the pathogen. The diagnosis of systemic infections is complex and involves clinical imaging, serology, and molecular tests in addition to microscopy and culture, and has been reviewed elsewhere (5).

This review outlines the different types of fungal infections that manifest in the skin, how cutaneous fungal infections are identified in a microbiology laboratory, and the role of dermatopathology in the diagnosis of fungal skin infection.

## Laboratory Detection of Fungal Infection

The traditional methods to detect fungal infection in a microbiology laboratory are microscopy and culture (6). The presence of a fungal infection can be confirmed by the observation of fungal structures in tissue by microscopic examination. This process involves placing a piece of skin, nail or tissue in a potassium hydroxide solution on a slide to soften. Once soft the preparation is squashed to make it as thin as possible as it is easier to detect fungal elements in a thin sample. Samples of hair or scalp are not squashed prior to initial microscopic examination as the arrangement of fungal elements within the hair can give useful information. A fluorescence stain such as calcofluor can be added to the sample to enhance visualization under filtered UV light (7, 8). The identity of the causative organism is determined following growth on culture plates (9). Although molecular diagnostics are available for life threatening infections and to detect specific panels of fungi (mainly used in larger laboratories), the microscopic detection of fungal structures in tissue remains a gold standard in diagnosis (6).

## Types of Fungal Infection

Fungal infections can be divided into three categories: superficial and cutaneous, subcutaneous, and systemic (see Table 1). Superficial and cutaneous infections may occur following contact with an infectious source, or from a change in the host or the skin flora. These infections are unsightly, uncomfortable, some are contagious, and they can affect anyone (10).

**TABLE 1:** Types of fungal infections.

Type of mycosis	Tissue type	Tissue involvement	Sources, route/risks of infection
Superficial and cutaneous	Skin, hair and nail	Skin infections confined to epidermis	Human, animal, soil
			For mould and environmental yeasts—contact with infected material, minor abrasion
			For commensal yeast species—changes in host susceptibility, underlying disease
Subcutaneous	Skin	Penetration of the dermis and subcutaneous tissue	Traumatic inoculation, fungi are environmental
Systemic	Blood, lungs, internal organs, dissemination possible	Dissemination from internal infection into the skin may be possible	Opportunistic infections—major risk is immune suppression, underlying disease, hospitalisation
			Endemic dimorphic infections—inhalation of fungal spores, immune suppression

Subcutaneous infections result from penetrating traumatic inoculation deep into the skin by objects contaminated with fungi from the environment. They can progress slowly and can cause significant disfigurement if left untreated (10).

There are two types of systemic fungal infections: opportunistic infections are caused by fungi that have a worldwide distribution which can cause significant illness in debilitated hosts, particularly the immune suppressed. These infections can be caused by commensal flora (such as *Candida* species) or from the environment. In contrast the endemic dimorphic fungi are highly virulent, are mostly acquired by the inhalation of fungal spores, and can cause disease in immune competent and immune compromised people (10).

## Superficial/Cutaneous Mycoses

**FIGURE 1:**
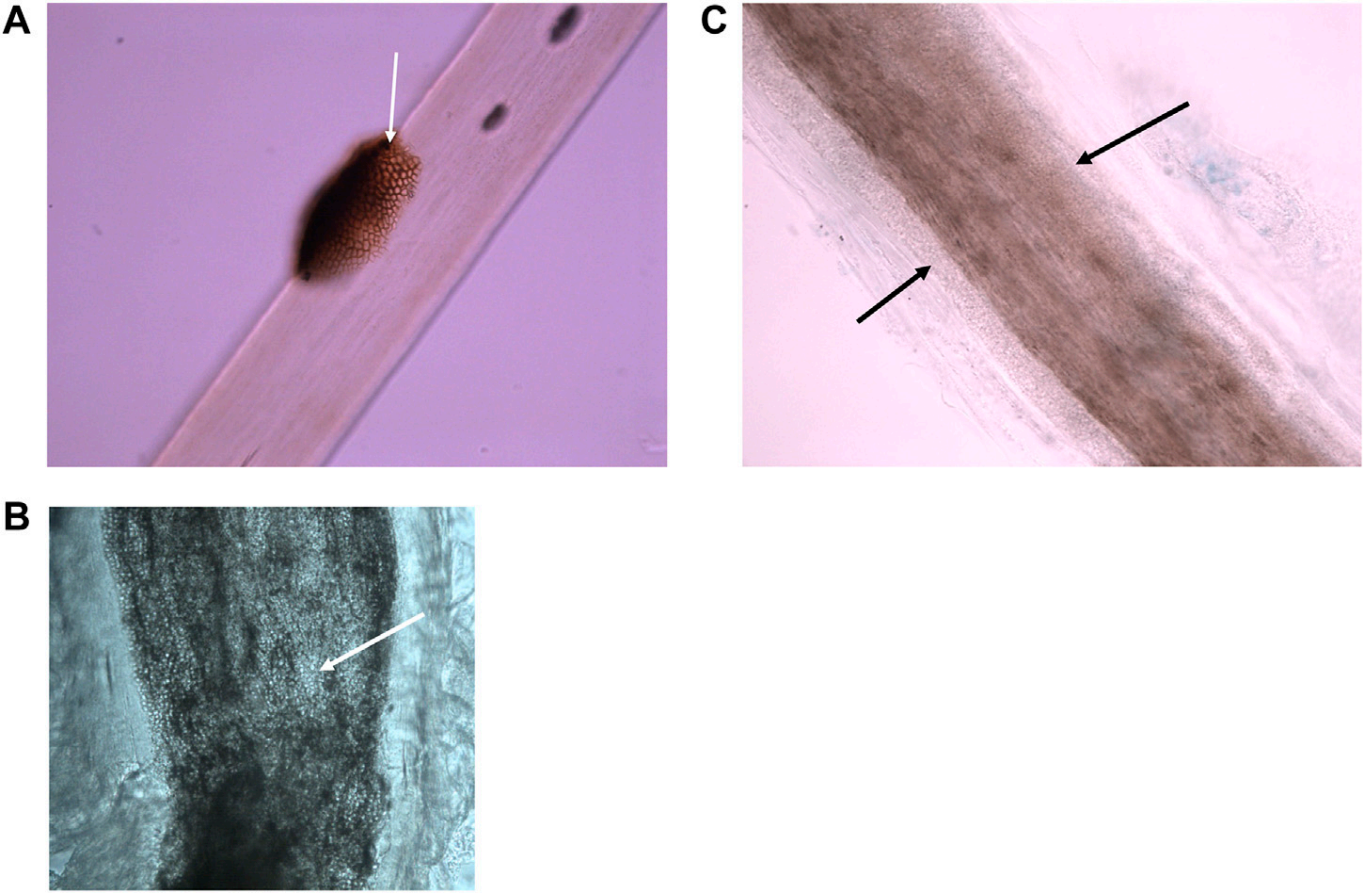
Microscopic appearance of superficial fungal hair infections using bright field microscopy. **(A)** Black piedra—a dark nodule containing pigmented hyphae is located on the surface of a hair shaft. (Photographed with a ×40 lens). **(B)** Endothrix dermatophyte infection—hyphae and arthroconidia (spores) found within the borders of the hair shaft (photographed using a ×40 lens). **(C)** Small spored ectothrix dermatophyte infection—hyphae and arthroconidia found surrounding the hair shaft forming a sheath. (Photographed using a ×40 lens).

**FIGURE 2:**
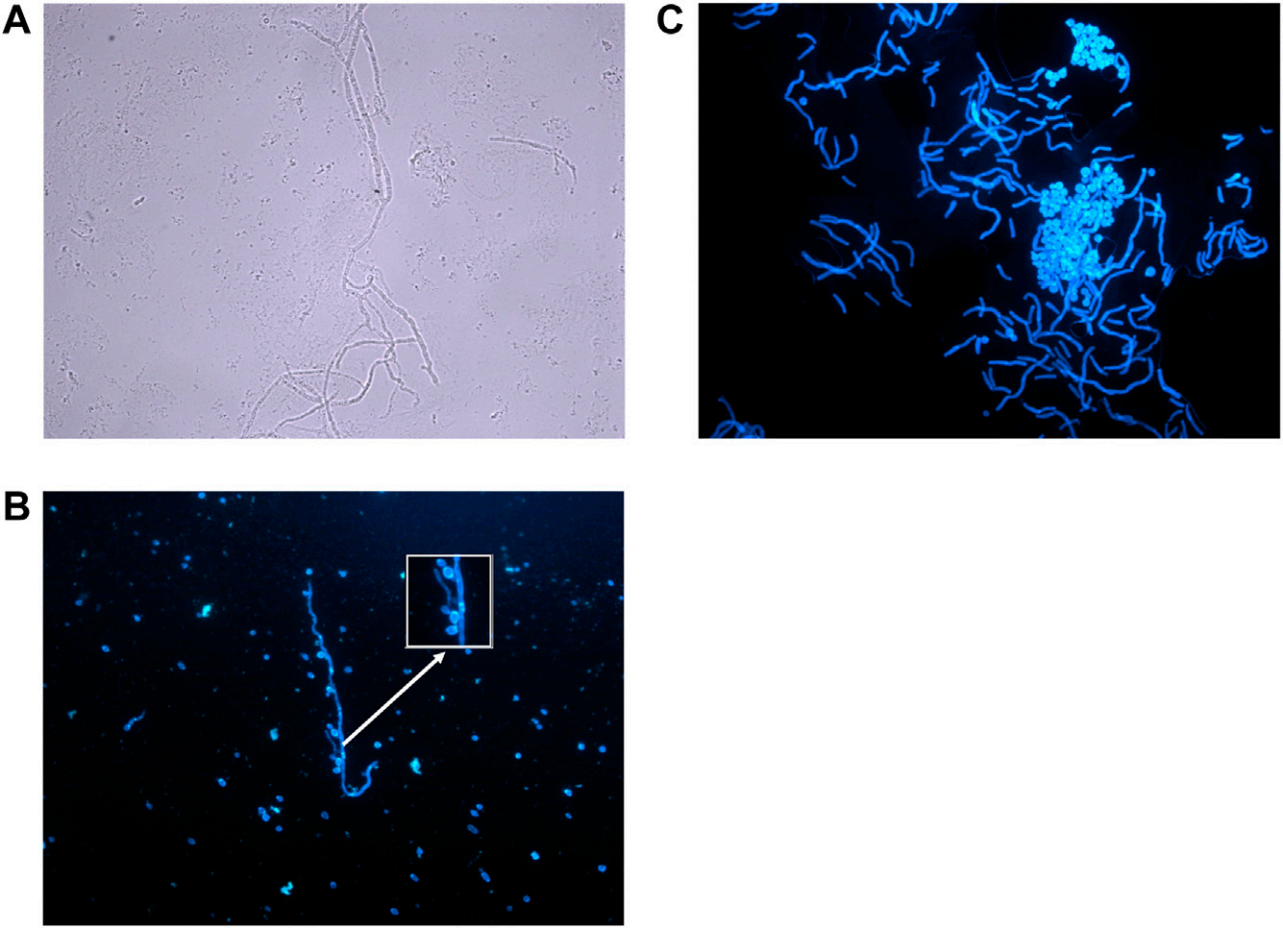
Microscopic appearance of fungal skin and nail infections. **(A)** Mould hyphae—Colourless (hyaline), septate, even sided, branching hyphae. (Photographed using a ×40 lens). **(B)** *Candida* species—Small budding yeasts (3–5 μm) that bud from any point on the cell. In this diagram budding yeasts are also attached to a hypha/pseudohypha (stained with calcofluor and photographed using ×40 lens). **(C)** Pityriasis versicolor—Thin walled, short hyphal filaments that fragment. Thick walled, round to oval budding yeasts with prominent bud scars (stained with calcofluor, photographed using ×40 lens).

Superficial and cutaneous mycoses are fungal infections of the skin, hair, and nails and are generally confined to the epidermis of the skin (10). These infections can have characteristic clinical presentations (11–15) which enable diagnosis with the aid of microscopy and culture (Table 2; Figures 1, 2). Rare deeper infections can arise when infection penetrates *via* infected follicles presenting as pustules, papules or nodules, as occurs in Majocchi’s granuloma (4, 11).

**TABLE 2:** Superficial/cutaneous mycoses, causative organisms, and common clinical features (4, 10, 11, 15).

Tissue	Infection	Cause	Clinical features
Hair	Black Piedra	*Piedraia hortae*	Dark hard nodules on the hair shaft
	White Piedra	*Trichosporon* yeasts	Soft white nodules on the hair shaft
	Tinea capitis	Dermatophytes mostly *Trichophyton* and *Microsporum* species	Scaling, itch, erythema, hair loss, broken hair at or just above the scalp surface, can lead to an inflammatory kerion lesion
Nail	Tinea unguium	Dermatophytes—mostly *Trichophyton* and Epidermophyton species	Discolouration, streaking, hyperkeratosis
	Onychomycosis	Generic term for any fungal cause—mould or yeast	Discolouration, streaking, hyperkeratosis, paronychia, onycholysis
	*Candida*	*Candida* species	Paronychia, onycholysis, discolouration
Skin	Tinea	Dermatophyte species	Scaling, erythema, presence of a raised leading edge with some healing behind. Itch
	*Candida*	*Candida* species	Scaling, erythema, maceration, satellite lesions forming away from lesion edge, may be sore
	Pityriasis versicolor	*Malassezia* species	Scaling, hypo/hyper pigmentation. Itch
	Tinea nigra	*Hortea werneckii*	Scaling, dark discolouration

Infections that can arise from the host commensal flora are caused by *Candida* and *Malassezia* yeasts, therefore risks for infection include changes in the host susceptibility, underlying disease, and integrity or damage of the tissue (12, 14). *Candida albicans* is a commensal of the gastrointestinal tract and is the commonest cause of *candida* skin and nail infection. Superficial *candida* skin infection tends to occur where the skin is moist such as the axillae, groin, perineum, web spaces, and other skin folds where the conditions permit the yeast to colonise and proliferate (14) (Figure 2).

*Malassezia* species are common skin commensal yeasts that prefer regions with greater skin lipid content such as the chest, back, face and scalp. The genus contains 17 species, 10–12 have been identified from the human skin flora, and many have been associated with skin infections (16). One of the commonest skin infections is pityriasis versicolor and microscopic examination of the infected skin shows characteristic broad short hyphae, which have been described as having an appearance resembling spaghetti and meatballs (6) (Figure 2).

Dermatophyte moulds cause infections known as ringworm or tinea and these can be contagious. Dermatophytes are found worldwide and there are species that have evolved to live on humans, on specific animals, and in soil, so infection requires contact with a source and probably mild abrasion or prolonged contact to initiate the infective process (17). Microscopic examination cannot determine the causative species so identification relies on growth of the fungus on culture. Knowledge of the identity of the fungus can indicate a likely source of infection and any actions that can be taken to reduce the risk of re-infection. If a scalp sample contains intact infected hairs it can be possible to determine the type of dermatophyte infection: for example, endothrix infections are passed person to person and are caused by *Trichophyton* species, whereas ectothrix infections can be from a human, animal or soil sources (8) (Figure 1).

Dermatophytes are considered to be pathogens as their isolation from tissue is associated with the presence of clinical infection, however, common environmental fungi can be isolated from skin, hair and nail as contaminants of culture plates (17). Interpretation of the isolation of an environmental fungus from a non-sterile site is complex and requires the detection of fungal structures in the tissue by microscopic examination for the fungus to be considered for significance (7).

The fungi that cause the hair infections known as black and white piedra are more common in hot humid environments. Risk factors for infection in these environments are colonisation of the hair, particularly if it is kept tied or braided (18). The infection presents as a nodule on the hair shaft and the nodule colour and hardness form part of the clinical description of the condition. Microscopic examination confirms the type of infection while culture enables the pathogen to be identified (Figure 1).

## Importance of Histology for the Diagnosis of Superficial and Cutaneous Fungal Infection

A failure to diagnose a superficial mycosis can be due to the quality and amount of material provided for testing (4). Many factors can alter the clinical appearance of a fungal infection making diagnosis difficult. For example, a history of treatment with steroids can alter the clinical appearance by reducing scaling and itch, a condition known as tinea incognito (10). Similarly partial treatment with an antifungal can prevent good samples being taken for microbiology assessment by reducing the scaling of the lesion. Patients with underlying diseases can present with an atypical appearance or an atypical distribution of lesions (4). In these circumstances biopsy samples taken for histopathology analysis should provide a diagnosis, although the causative species cannot be determined by this process.

For histological examination skin, hair and nail samples are routinely embedded, sectioned, and stained with Hematoxylin and Eosin (H&E) (19). H&E provides a background stain and highlights the presence of nucleic acids within cells by staining nucleic acids blue and the cytoplasm pink (20). Fungal structures that lack pigment (hyaline) can be difficult to detect as the stain does not bind to the cell wall, therefore, special stains can be used to highlight the presence of fungal cells: Periodic Acid-Schiff (PAS), Grocott and Gomori Methenamine Silver (GMS), and Fontana-Masson stain (Table 3). These stains can highlight the presence of non-fungal materials as well, so a histopathology diagnosis of a fungal infection requires careful inspection of the morphology, surrounding tissue reactions, and interpretation of clinical information (20).

**TABLE 3:** Histology stains used in the diagnosis of fungal infections (20).

Stain	Appearance of fungus in tissue	Reaction in tissue
H & E	Cytoplasm pink	Stains nucleic acids blue/purple. Stains proteins in cytoplasm, membranes, etc., pink. Useful to highlight inflammatory processes in tissue
	Nucleus blue	
	Cell wall colourless	
PAS	Cell wall pink to red to purple	Stains carbohydrates, polysaccharides, glycogen, mucoproteins, and mucins in tissue
GMS	Cell wall dark brown to black	Primarily used to bind to carbohydrate in cell walls of fungi. Tissue usually stained green
Fontana Masson	Stains melanin deposits brown to black	Stains melanin deposits in tissue

The appearance of mould and yeast structures in tissue are similar when observed in wet preparations and in histology sections, however, there are some conditions where histological examination is superior. For example, skin folliculitis caused by *Malassezia* yeasts is diagnosed using clinical criteria and histological examination of a biopsy (12). The condition is caused by an accumulation of these yeasts within follicles which are surrounded by inflammation. A potassium hydroxide mount would not demonstrate the location of the yeasts as the process is destructive and the skin structures would be destroyed, and only histopathology can demonstrate the presence of inflammation around the follicles.

## Subcutaneous Infections

**FIGURE 3:**
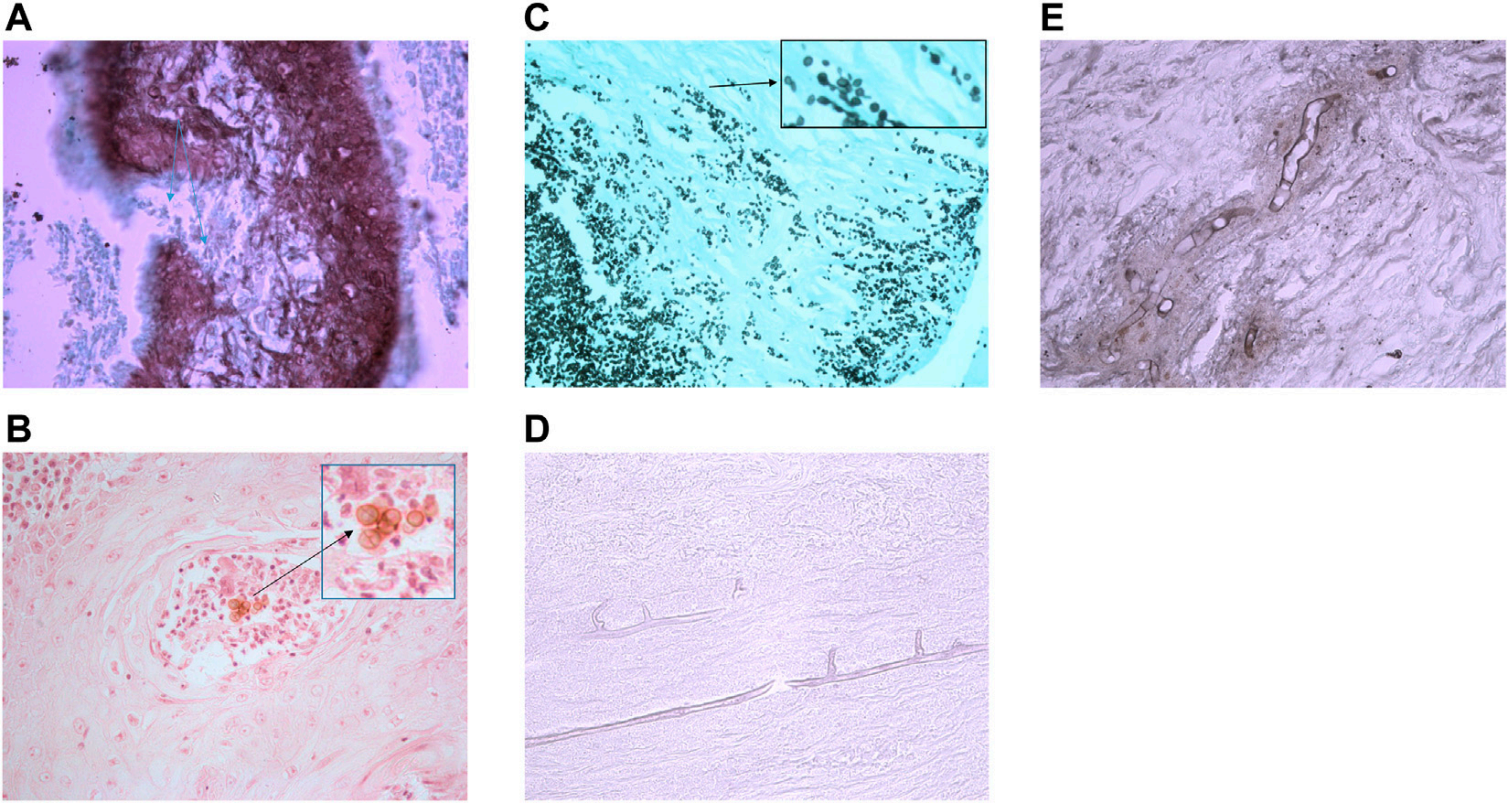
Histology sections of subcutaneous fungal infections demonstrating characteristic morphology for each disease type. **(A)** Eumycetoma grain in tissue stained with GMS. Interior of grain contains brown/stained hyphae that have been sectioned longitudinally and horizontally resulting in various shaped fungal structures (photographed using a ×40 lens). **(B)** Pigmented muriform bodies in a biopsy from a chromoblastomycosis lesion stained with H&E. (Photographed using a ×40 lens). **(C)** Sporotrichosis stained with GMS. In tissue *Sporothrix* produces yeast-like budding with characteristic elongated buds. (Photographed using a ×40 lens). **(D)** Mucormycosis of tissue stained with PAS shows wide, thin-walled hyphae with few septa. (Photographed using a ×40 lens). **(E)** *Conidiobolus* infection stained with GMS shows wide, thin-walled hyphae with few septa, but these are surrounded by inflammatory material (photographed using a ×40 lens).

Subcutaneous fungal infections result from traumatic inoculation deep into the skin and are mostly due to environmental moulds. Identification of the pathogen requires culture, but histology can provide important diagnostic information, especially when routine microscopy and culture are negative (Table 4; Figure 3) (4, 19).

**TABLE 4:** Subcutaneous fungal infections (4, 10, 15, 21, 22).

Disease	Common causes	Clinical presentation	Histology
Mycetoma	*Madurella mycetomatis Trematosphaeria grisea*, *Falciformispora senegalensis, Phialophora, Scedosporium*, *Fusarium species*, *Aspergillus nidulans, Sarocladium*, (A*cremonium*)	Chronic infection of skin, subcutaneous tissue and bone. Nodule forms an abscess and drains to the surface *via* sinus, discharge contains grains. Deformity and disability considerable. Important to distinguish bacteria from fungal cause	Presence of a grain surrounded by an inflammatory reaction. Eumycetoma grains contain narrow hyphae—pigmented (dark grain) or hyaline (pale grain)
Sporotrichosis	*Sporothrix schenckii, S. brasiliensis*, *S. mexicana*, *S. globose, S. luriei*	Cutaneous infection: initial nodule forms a granuloma or ulcer with satellite lesions at the edges, secondary nodules appear along course of draining lymphatics, commonly limbs	Oval to elongated (cigar shaped) yeasts with budding from a narrow base, asteroid bodies
Chromoblastomycosis	*Fonsecaea pedrosoi*, *F. compacta*, *Cladophialophora carrionii*, *Phialophora verrucosa*	Chronic, slow growing warty nodule to plaque, autoinoculation results in satellite lesions. Elephantitis	Epidermal hyperplasia, granulomatous response, transepidermal elimination of debris, presence of round thick walled fungal cells (muriform bodies)
Phaeohyphomycosis	*Exophiala jeanselmei, E. dermatitidis, E. spinifera, Phaeoacremonium parasiticum, Phialophora verrucosa, Alternaria alternata, Bipolaris* species*, Scedosporium* species*, Lomentospora prolificans, Curvularia lunata*	Subcutaneous cysts, abscess, verrucous plaque. Immune suppression and transplant are risk factors	Pseudoepitheliomatous hyperplasia, inflammatory infiltrate, presence of septate pigmented hyphae or hyphal fragments
		Some species can cause systemic infections	
Hyalohyphomycosis	*Aspergillus* species	Varied: verrucous, erythematous macules, papules and indurated nodules, ulceration	Inflammation, granulomatous reactions, narrow, regularly septate hyphae
	*Fusarium* species	Some species can cause systemic infections	
	*Scedosporium* species		
	*Acremonium* species		
	*Paecilomyces/Purpureocilllium* species		
Mucormycosis	*Rhizopus* species	Necrosis, ulceration, swelling, inflammation, rapid progression	Wide, thin walled, pauciseptate hyphae, Branching may be at right angles. Hyphae may appear distorted. Little inflammation surrounding hyphae
	*Lichtheimia corymbifera*		
	*Rhizomucor* species		
	*Mucor* species		
Entomophthoromycosis	*Basidiobolus ranarum Conidiobolus coronatus*	Slowly progressing subcutaneous swelling: *Conidiobolus* mainly affects the face and nasal passages	Wide, thin walled, pauciseptate hyphae, Branching may be at right angles. Hyphae may appear distorted. Surrounded by granulomatous inflammatory reaction
		*Basidiobolus* mainly affects limbs	
Lobomycosis	*Lacazia loboi*	Papule slowly progresses to form nodules or plaques with keloid like appearance. Older lesions can be crusted	Oval yeast-like cells connected in short chains by a tubular structure

Mycetoma can be caused by filamentous bacteria (actinomycetoma) or by fungi (eumycetoma) (4, 23). Pieces of the infective organisms become encased in an inflammatory matrix that forms grains and these can be expressed from subcutaneous abscesses *via* a sinus tract, or can be seen in the tissue from a deep subcutaneous biopsy. Analysis of the grains distinguishes the type of mycetoma and enables appropriate empirical antifungal or antibacterial therapy to be commenced. The simple distinction between actinomycetoma from eumycetoma is important as culture of the grains may fail or yield fungi that lack typical features making them difficult to identify unless there is access to a centre that performs molecular diagnostic testing (24, 25).

*Sporothrix* species cause a subcutaneous infection following traumatic inoculation. Subcutaneous infection in immune competent patients can have a low fungal load, making detection of the characteristic elongated budding yeasts difficult by direct microscopy (26). Histology is more sensitive as it detects the areas of inflammation where the fungus might reside, and with the use of serial sections and special stains can detect the presence of scanty yeast-like cells and asteroid bodies which are present in many sporotrichosis infections (20).

Chromoblastomycosis is a slowly progressing, chronic, pigmented fungal infection. This is characterised by the presence of pigmented, multiseptate, round, thick-walled cells sometimes referred to as muriform bodies (4, 19, 27). These cells are not detected in other subcutaneous pigmented fungal infections which are grouped under the heading of phaeohyphomycosis, and are more hyphal (19).

Hyalohyphomycosis is the subcutaneous (or systemic) infection with a non-pigmented fungus. Many species can cause hyalohyphomycosis but the species cannot be determined by histology. All species would demonstrate narrow, regularly septate branching hyphae (20).

Mucormycosis is a subcutaneous infection that follows trauma to the skin from penetrating injuries, scratches, or by contamination of burns or injuries, and can cause systemic infection following inhalation of spores mainly in immune compromised patients (28). The main causes of this infection belong to a group known as the mucoraceous moulds, and these fungi grow rapidly in the tissue and have a predilection for blood vessel invasion. These moulds exist worldwide in the environment and differ from other types of moulds as they have broader hyphae and far fewer septa within the hyphae. As a result they often have rather distorted appearances when sectioned and appear creased or with an unusual shape.

Although the Entomophthorales moulds may appear similar in appearance in the tissue to the mucoraceous moulds, they cause clinically very different infections (subcutaneous swellings, not angioinvasive) and histologically the broad aseptate hyphae are surrounded by an inflammatory reaction (28).

Lobomycosis is a cutaneous to subcutaneous infection caused by the *Lacazia loboi* and this environmental fungus is found in the tropical climates of Central and South America, and most often affects people in rural communities. The fungus does not grow on culture so diagnosis is based only on clinical suspicion and histology (29).

## Systemic Fungal Infections That Can Disseminate to the Skin

**FIGURE 4:**
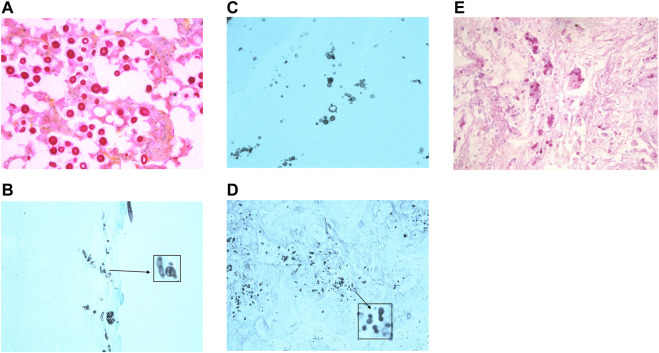
Histology images of some causes of systemic fungal infection. **(A)** *Cryptococcus* yeasts in tissue stained with mucicarmine to demonstrate the presence of the red stained mucopolysaccharide capsule. (Photographed using a ×40 lens). **(B)** Talaromycosis histology section stained with GMS shows yeast cells with a central division, as these cells divide by fission and not by budding. (Photographed using a ×40 lens). **(C)** Paracoccidioidomycosis (stained with GMS) is characterised by the presence of multipolar budding yeast cells. (Photographed using a ×40 lens). **(D)** Histoplasmosis stained with GMS to show the small budding yeasts. (Photographed using a ×40 lens). **(E)** Blastomycosis stained with PAS shows large thick walled yeasts that bud from a broad base. (Photographed using a ×40 lens).

Systemic opportunistic fungal infections occur in significantly debilitated hosts including those with immune suppression, transplant recipients, surgery, hospitalization, broad spectrum antibiotic treatment, or in patients with other significant underlying disease (10). It is possible for systemic fungal infections to disseminate *via* the bloodstream and produce lesions in the skin (4). Histology of a biopsy can detect the presence of a fungal cause and provide information on the type of underlying infection (Table 5; Figure 4).

**TABLE 5:** Types of systemic infection and their features on histology sections (19, 20, 21, 22).

Disease	Causative organism	Clinical features	Histological features
Candidiasis	*Candida albicans, Candida* species	Invasive and disseminated infection occurs in seriously ill and immune compromised patients	Budding yeast cells (3–5 μm), hyphae and pseudohyphae (constriction occurs where there are septa). *C. glabrata* does not produce hyphae
Cryptococcosis	*Cryptococcus neoformans, Cr. gatii*	Pulmonary infection, dissemination in the immune compromised, meningitis in AIDS.	Small yeasts (4–10 μm) budding from a narrow base, mucopolysaccharide capsule stains well with mucicarmine. Fontana Masson stain useful for acapsular variants
Histoplasmosis	*Histoplasma capsulatum, H. capsulatum var. duboisii*	Pulmonary disease, dissemination in immune compromised. *H. duboisii* disseminates to skin and bone, Africa	Small (2–4 μm) round to oval yeasts, budding from a narrow base, may be clustered intracellularly. *H. duboisii* are larger (8–15 μm) budding yeasts
Blastomycosis	*Blastomyces dermatiditis, B. gilchristii*	Pulmonary disease. Dissemination possible	Large thick walled yeasts (8–15 μm) that bud from a broad base
Paracoccidioidomycosis	*Paracoccidioides brasiliensis, P. lutzii*	Pulmonary disease. Dissemination especially mucocutaneous	Large yeast cells (15–30 μm) with multiple budding—ship’s wheel appearance
Coccidioidomycosis	*Coccidioides immitis, C. posadasii*	Acute or chronic pulmonary disease, lung cavities form, dissemination possible in immune competent and immune compromised	Thick walled spherules (10–100 μm) containing endospores (2–5 μm) in different stages of development, granulomatous reaction
Talaromycosis	*Talaromyces marneffei*	Pulmonary infection, mainly immune compromised. Dissemination	Small oval or curved yeast like cells that divide by transverse fission forming a thick septum. May be clustered inside macrophages
Emergomycosis (Emmonsiosis)	*Emergomyces (Emmonsia) p*asteurianus, *Es. africanus, Es. canadensis, Es. orientalis, Es. europaeus*	Pulmonary infection with dissemination, immune compromised hosts	Yeast-like cells (2–7 μm)

*Candida* species are commensal to the human gastrointestinal tract, and are found in low numbers on the skin, mouth and vaginal tract. *C. albicans* is the commonest cause of human infection, although there has been a rise in systemic infections caused by non- *C. albicans* species (1). Superficial infection occurs when there is a disturbance in the competing bacterial flora allowing the yeasts to proliferate, such as the use of broad spectrum antibiotics or occlusion, or due to underlying disease, for example, diabetes or HIV. Systemic infection occurs in patients where the yeasts escape from their normal location by the colonisation of indwelling lines or other implants, or due to the effects of broad spectrum antibiotics, immune suppression, neutropenia, cancer, or surgery (14, 30). *Candida* infection in tissue can present with a variety of morphologies: budding yeasts, hyphae, and pseudohyphae (6, 8, 19) (Table 5).

*Aspergillus* and *Fusarium* species are common environmental moulds that can cause systemic infection in patients with neutropenia, haematological malignancies, and in immune suppressed patients usually by inhalation of airborne spores. These moulds can disseminate *via* the bloodstream and can cause skin lesions (20). It is not possible to distinguish the species by microscopic appearance in tissue (19, 31) (Table 4). As there can be important differences in the treatment of infections caused by different species histology reports generally describe the type of fungal structures seen, for example, hyalohyphomycosis, rather than suggesting a causative organism (19, 31).

*Cryptococcus neoformans* and *Cr. gatii* are environmental yeasts that can cause pulmonary infection by inhalation of spores or yeasts, and in immune suppressed patients dissemination can occur to skin and other tissue (32). Cryptococcal meningitis is a significant risk in AIDS patients when their CD4 count falls below 100 cells per µl (32). A major characteristic of these yeasts is the presence of a mucopolysaccharide capsule which on microscopy gives the appearance of a clear halo surrounding the small budding yeasts in tissue (6). The capsule can be highlighted in microbiology wet preparations using India ink, and in histology sections using the mucicarmine stain. Acapsular variants can be confused with *Histoplasma* or *Candida* yeasts, but use of mucicarmine to detect remnants of capsule, or staining with Fontana Masson to detect melanin in the cryptococcal cell walls, will assist in differentiation of these organisms in tissue (20, 32).

The diseases histoplasmosis, paracocciodiodomycosis, blastomycosis, coccidioidomycosis, and talaromycosis are caused by highly infectious endemic dimorphic moulds. These fungi are limited in location geographically and environmentally and exist in the environment as a mould, however, when they infect the human host they demonstrate a different more yeast-like appearance (33). The areas where infections are endemic are listed in Table 6, and people who have a travel history to these regions may become infected. All of these infections are acquired by inhalation of spores that leads to a pulmonary infection. Dissemination to the skin is possible in some patients, usually those that have not received treatment or have immune suppression (4, 33). Histology sections of these fungi may demonstrate a distinctive morphology, but culture of the pathogen and serology is always recommended to confirm the diagnosis. Detailed descriptions of the histological appearance of these infections and how to distinguish them from unusual presentations of other infections can be found in the review by Guarner and Brandt (20).

**TABLE 6:** Locations of endemic dimorphic fungal infections (4, 21, 33, 34, 35).

Disease	Organism	Location
Histoplasmosis	*H. capsulatum*	States around the Mississippi and Ohio river valleys (eastern to central USA), smaller foci in Central (Mexico) + South America (e.g., Venezuala to Argentina), Africa, Asia (Thailand) and India, not in northern Europe
	*H. duboisii*	Found in Africa and between Tropics of Cancer and Capricorn, and also on Madagasgar
Coccidioidomycosis	*C. immitis*	California, USA
	*C. posadasii*	Texas, Arizona, Mexico, central and south America
Blastomycosis	*B. dermatitidis*	Areas prone to flood, USA, Canada, small foci in North and Central Africa and in India
Paracoccidioidomycosis	*P. brasiliensis, P. lutzii*	Central and South America, not Chile. Moist subtropical forested areas with high rainfall
Talaromycosis	*T. marneffei*	Southeast Asia, Thailand, Hong Kong, China
Emergomyces	*Es. p*asteurianus,	Europe (Italy, Spain, France, the Netherlands), Asia (China and India), Uganda, and South Africa
	*Es. africanus*	South Africa and Lesotho
	*Es. canadensis*	Canada (Saskatchewan), USA (Colorado and New Mexico)
	*Es. orientalis*	China
	*Es. europaeus*	Germany

Emergomyces (previously Emmonsia) infections usually start with a pulmonary infection and many disseminate to the skin (33). These fungi infect immunocompromised patients such as solid organ transplant recipients, HIV, and those receiving corticosteroid treatment and have been reported from South Africa, Canada, Germany, Israel, Italy, China (33–35). Dissemination to the skin occurs in 92%–96% of the patients examined (34, 35). Skin lesions were often clinically misdiagnosed as Karposi sarcoma, varicella infection or drug reactions, but the visualisation of the yeast-like cells on histology sections confirmed the presence of a fungal infection and that a systemic source should be investigated. Culture is required to confirm the cause of infection and distinguish it from other dimorphic fungal infections such as histoplasmosis which also presents as small budding yeasts in the skin (34, 35).

## Advanced Diagnostic Histological Techniques

Immunohistochemistry (IHC) is used to detect protein antigens in histology sections and is widely employed in the diagnosis of melanoma, other skin cancers, a variety of other skin diseases, and to detect the presence of some infections (36). To detect systemic fungal infection IHC stains are commercially available to detect *Aspergillus* species, *Candida* species and Mucormycete moulds, but the potential of cross-reaction with other fungi means this cannot be used to identify a specific organism (20, 37).

*In situ* hybridisation (ISH), uses a species-specific probe to detect fungal nucleic acids in tissue sections. This should be highly specific, but there are no commercially validated protocols and there have been reports of cross-reactions (37). The advantage of both IHC and ISH is that they maintain the structure of the tissue and the localisation of the fungal antigen or nucleic acid for histopathological examination. Both tests have greatest sensitivity when applied to samples where GMS or PAS staining has already demonstrated the presence of fungal infection and a panel of tests can be performed to indicate the likely genus of the infecting agent (20, 37).

Amplification of fungal DNA directly from a sample potentially provides a sensitive and specific method to diagnose fungal infection. Amplification from non-embedded fresh tissue is more reliable than from FFPE tissue due to the difficulties with extraction, DNA concentration and integrity, and the presence of inhibitors (20). The choice of target sequence for amplification requires careful consideration to ensure it can distinguish between many different species, and that there are sufficient sequences in public validated databases that are available for comparison (37). The internal transcribed spacer (ITS) regions have been used for fungal DNA barcoding and the ISHAM database has sequences for 421 fungal species, however, there are some fungi that cannot be adequately identified using this target alone and so additional genetic markers would need to be used (38). Amplification directly from FFPE tissue would also have to consider the possible presence of environmental contamination of histology laboratory materials, for example, the wax used for embedding is not stored or used under sterile conditions (37). Despite these difficulties the importance of histology in the diagnosis of fungal infections is recognised. Recent guidelines for the diagnosis of invasive fungal infections permit the use of PCR and sequencing to identify the fungus from tissue where fungal structures have been seen on histology (37). By following the ten criteria explained in the revised guidelines samples with histopathology evidence of fungal infection and a fungal identification using PCR and sequencing would fulfil the diagnosis of a proven invasive infection, and would be especially important in cases where culture fails to grow a pathogen. The guidelines also recommend this molecular approach where an endemic dimorphic fungal infection has been detected by histopathology and the microbiology laboratory has little experience with these pathogens.

## Conclusion

Histology provides a gold standard for the diagnosis of fungal infections from biopsies. Special stains such as PAS and GMS greatly enhance the detection of fungal elements making this a highly sensitive technique. Careful morphological examination of the tissue, in combination with a clinical history can indicate the type of fungal infection, but it cannot identify the causative species. Identification of the fungal species can indicate the most appropriate choice of treatment for the patient. Currently fungal identification still relies on culture, but molecular diagnostics are developing for the detection of subcutaneous and systemic pathogens directly from tissue samples.
